# Sulfated Hyaluronan Modulates the Functional Properties and Matrix Effectors Expression of Breast Cancer Cells with Different Estrogen Receptor Status

**DOI:** 10.3390/biom11121916

**Published:** 2021-12-20

**Authors:** Christos Koutsakis, Anastasia-Gerasimoula Tavianatou, Dimitris Kokoretsis, Georgios Baroutas, Nikos K. Karamanos

**Affiliations:** 1 Biochemistry, Biochemical Analysis and Matrix Pathobiology Research Group, Laboratory of Biochemistry, Department of Chemistry, University of Patras, 265 04 Patras, Greece; ckoutsakis@upatras.gr (C.K.); natasha.tavianatou@hotmail.gr (A.-G.T.); kokoretsisdimitris@gmail.com (D.K.); baroutasg@gmail.com (G.B.); 2 Foundation for Research and Technology-Hellas (FORTH)/Institute of Chemical Engineering Sciences (ICE-HT), 265 04 Patras, Greece

**Keywords:** sulfated hyaluronan, extracellular matrix, breast cancer, estrogen receptors, epithelial-to-mesenchymal transition, matrix metalloproteinases

## Abstract

Hyaluronan (HA) is an extracellular matrix glycosaminoglycan (GAG) that plays a pivotal role in breast cancer. While HA is the only GAG not normally substituted with sulfate groups, sulfated hyaluronan (sHA) has previously been used in studies with promising antitumor results. The aim of the present study was to evaluate the effects sHA fragments have on breast cancer cells with different estrogen receptor (ER) status. To this end, ERα-positive MCF-7, and ERβ-positive MDA-MB-231 cells were treated with non-sulfated HA or sHA fragments of 50 kDa. The functional properties of the breast cancer cells and the expression of key matrix effectors were investigated. According to the results, sHA attenuates cell proliferation, migration, and invasion, while increasing adhesion on collagen type I. Furthermore, sHA modulates the expression of epithelial-to-mesenchymal transition (EMT) markers, such as e-cadherin and snail2/slug. Additionally, sHA downregulates matrix remodeling enzymes such as the matrix metalloproteinases MT1-MMP, MMP2, and MMP9. Notably, sHA exhibits a stronger effect on the breast cancer cell properties compared to the non-sulfated counterpart, dependent also on the type of cancer cell type. Consequently, a deeper understanding of the mechanism by which sHA facilitate these processes could contribute to the development of novel therapeutic strategies.

## 1. Introduction

Breast cancer is characterized by high heterogeneity and constitutes one of the most commonly reported types of cancer globally [1]. Throughout disease development, the expression patterns of the estrogen receptors (ERs) are crucial for regulating breast cancer cell properties, morphology, as well the expression of several effectors associated with aggressiveness of the malignancy [2,3,4]. Depending on the ER status, breast cancer cells are categorized into ERα-positive, with epithelial characteristics and low metastatic potential, and ERα-negative, which exhibit higher metastatic potential and are associated with more aggressive phenotypes [5]. During breast cancer progression, cellular phenotypes can be altered due to epithelial-to-mesenchymal transition (EMT), which leads to loss of cell polarity and cell-to-cell junctions, thus facilitating cellular migration and metastasis [6,7]. As a result, the expression of epithelial markers, such as e-cadherin, is significantly downregulated, whereas the expression levels of mesenchymal markers, such as snail2/slug, are elevated [8,9,10]. Additionally, overexpression of matrix enzymes, such as matrix metalloproteinases (MMPs) accounts for the extensive reorganization of the extracellular matrix (ECM), further contributing to cell migration and invasion [11].

ECM is a complex, well-organized, three-dimensional network of macromolecules, which provides structural support to the cells and at the same time regulates their morphology and function [12,13]. One major component of ECM is the polysaccharide hyaluronan (HA), a linear non-sulfated GAG composed of disaccharide repeating units of d-glucuronic acid (GlcA) and *N*-acetyl-d-glucosamine (GlcNAc) [13]. HA is renowned for its role and participation in various physiological processes, like embryogenesis, wound healing and inflammation, as well as being linked to tumor initiation and development [14,15,16]. After binding to its receptors, HA can induce intracellular signaling pathways, thus regulating cell behavior. The most well-characterized HA receptor is CD44, which is implicated in various cellular processes [17,18,19]. Three enzymes are responsible for HA synthesis, the HA synthases HAS1–3, each producing HA of different sizes [20,21,22]. Moreover, HA degradation into low molecular fragments is carried out by the action of the specific hyaluronidases [23].

Depending on its molecular size, HA exerts different biological functions and regulates specific cellular behavior. For instance, HA fragments of <10, 30, and 200 kDa affect breast cancer cells’ functional properties and morphology, as well as the expression patterns of several molecules in a size-dependent manner [24,25]. Moreover, high molecular weight (HMW) HA is known for its anti-inflammatory, anti-angiogenic, and anti-proliferative properties [26]. Although elevated levels of endogenous HA have been linked with an aggressive phenotype in breast cancer cells, several studies have reported that low molecular weight (LMW) HA (100–300 kDa) can attenuate cancer cell growth and metastasis [27,28,29]. Recently, the effects of modified HA molecules have come under focus. These molecules have undergone modifications on their structure, such as the addition of sulfate groups. The inhibitory role of sulfated HA (sHA) in cancer has been shown in prostate cancer cells, where it attenuates the activity of HYAL1, decreasing cancer cell proliferation and invasion [30]. Moreover, the antitumor capabilities of sHA have also been shown in pre-clinical models of bladder cancer, where sHA fragments inhibited the proliferation, migration and invasion of cancer cells, as well as angiogenesis [31]. Together, these observations suggest the potential use of HA fragments as novel therapeutic approaches in malignancies. However, studies regarding the direct comparison between the effects exerted by non- and sulfated HA of the same molecular size are not available, according to our knowledge.

The aim of the present study was therefore to investigate the role of sHA on breast cancer cells with different ER status. To this end, non-sulfated 50 kDA HA and sHA fragments of the same molecular size were used to treat two breast cancer cell lines of different ER status: the less invasive ERα-positive MCF-7 and the highly aggressive ERα-negative/ERβ-positive MDA-MB-231 cells. Here, we demonstrate that sHA fragments regulate the functional properties of breast cancer cells and modify the expression of EMT markers, as well as key matrix effectors, such as MMPs and hyaluronan synthases.

## 2. Materials and Methods

### 2.1. HA and sHA Fragments

The HA fragments of 50 kDa were isolated at Fidia Farmaceutici S.p.A as described in a previous study of our research group [25]. The sulfated HA (MW = 50 kDa, degree of sulfation: 2.6) was kindly provided by Fidia Farmaceutici S.p.A (Abano Terme, Italy).

### 2.2. Cell Cultures and Reagents

The MCF-7 (ERα-positive, low metastatic potential) and MDA-MB-231 (ERα-negative/ERβ-positive, high metastatic potential) breast cancer cell lines were obtained from the American Type Culture Collection (ATCC, Manassas, VA, USA). The cells were cultured at 37 °C in a humidified atmosphere of 5% CO_2_ and 95% air. The cell culture medium used was Dulbecco’s Modified Eagle Medium (DMEM, LM-D1110/500, Biosera, Nuaillé, France), supplemented with 10% fetal bovine serum (FBS, FB-1000/500, Biosera, Nuaillé, France), antimicrobial agents (100 IU/mL penicillin, 100 μg/mL streptomycin, 10 μg/mL gentamycin sulfate and 2.5 μg/mL amphotericin B), 1 mM sodium pyruvate, and 2 mM L-glutamine (Biosera, Nuaillé, France). The cells were harvested using trypsin-EDTA 1× in PBS (LM-T1706/500, Biosera, Nuaillé, France) at approximately 80–85% cell confluency. All experiments were conducted in serum-free conditions using three separate biological replicates. The cytostatic agent cytarabine was purchased from Sigma-Aldrich (Saint Louis, MO, USA). All other chemicals used were of the best commercially available grade.

### 2.3. Proliferation Assay

MCF-7 and MDA-MB-231 cells were seeded on 96-well plates at a density of 7500 and 5000 cells per well respectively. After a 24 h incubation in complete cell culture medium, the cells were serum starved overnight. The HA and sHA fragments were added at a concentration of 200 μg/mL and the cells were incubated for an additional 24 h. In order to evaluate the effects on breast cancer cell proliferation a crystal violet assay was performed, as described in a previous study [32]. The cells were washed twice with PBS and stained with 0.5% (*w*/*v*) crystal violet in 20% methanol/distilled water solution. After a 20 min incubation on a bench rocker, the staining solution was aspirated, and the cells were washed three times with distilled water. The plates were left at room temperature to air dry overnight. The next day, methanol was added to solubilize the dye, followed by a 20 min incubation on a bench rocker. Finally, the optical density of each well was measured at 570 nm using a TECAN photometer.

### 2.4. Wound Healing Assay

MCF-7 and MDA-MB-231 cells were seeded on 12-well plates at a density of 3 × 10^5^ and 2.5 × 10^5^ cells per well respectively. The cells were cultured in complete cell culture medium for 24 h and were serum starved overnight. The next day, the cell monolayer was wounded by scratching with a sterile 100 μL pipette tip. The wells were then washed twice with PBS to remove the detached cells. To minimize possible contribution of cell proliferation to the migration results, serum-free medium containing the cytostatic cytarabine (10 μΜ) was added and remained until the end of the assay. After a 40 min incubation, the HA and sHA fragments were added at a final concentration of 200 μg/mL. The wound closure was captured at two time points of 0 and 24 h using a digital camera connected to a phase-contrast microscope. The cell migration was determined by quantification of the wound surface area difference between the two time points using image analysis (Fiji v1.52p) [33].

### 2.5. Collagen Type I Cell Adhesion Assay

MCF-7 and MDA-MB-231 cells were seeded on 6-well plates at a density of 3 × 10^5^ and 2.5 × 10^5^ cells per well respectively. The cells were cultured in complete cell culture medium for 24 h and were serum starved overnight. The next day, the HA and sHA fragments were added at a final concentration of 200 μg/mL in serum-free medium. Meanwhile, 96-well plates were prepared with 40 μg/mL collagen type I in PBS and were stored at 4 °C overnight. The next day the solution was aspirated, the plates were washed twice with PBS, and blocked with 1% BSA in PBS for 30 min. After a 24 h incubation, the cells in the 6-well plates were harvested using 4 mM EDTA in PBS, before being centrifuged and resuspended in serum-free medium containing 0.1% BSA. The cells were then seeded in the pre-coated collagen type I 96-well plates at a density of 2 × 10^4^ per well for the MCF-7 and 1 × 10^4^ cells per well for the MDA-MB-231. After seeding, the cells were incubated at 37 °C for 40 min to adhere to the collagen type I. Subsequently, the cells were washed twice with PBS to remove the non-adherent ones. To determine the adhesion rate, the adherent cells were stained following the crystal violet assay, as described above (Section 2.3).

### 2.6. Collagen Type I Invasion Assay

To evaluate the HA and sHA fragments effects on breast cancer cells invasive capacity, a collagen type I invasion assay was used. The cells were cultured in complete cell culture medium for 24 h and were serum starved overnight. A collagen type I solution was prepared as follows: 5 volumes of CMF-HBSS were mixed with 2.65 volumes of complete medium, 1 volume of MEM 10×, 1 volume of 0.25 M NaHCO3, 0.3 volumes of 1 M NaOH and 4 volumes of collagen type I (stock concentration 5 mg/mL). The final concentration of collagen type I was 1 mg/mL. The solution was spread homogeneously in 12-well plates and the plates were left to gellify at 37 °C, 5% CO_2_ for 1 h. The cells were then seeded in the plates at a density of 6 × 10^4^ cells per well. The HA and sHA fragments were added at a final concentration of 200 μg/mL and the cells were incubated for 24 h, after which, images were taken using a digital camera connected to a phase-contrast microscope. The quantification was performed using an image analysis software (Fiji v1.52p), as described in a previous study [34].

### 2.7. Wound Healing and Adhesion Assays Using Hyaluronidase Pre-Treatment

The processes followed were the same as the wound healing and adhesion assays (Section 2.4 and Section 2.5, respectively), with the distinctive step of adding hyaluronidase from Streptomyces hyalurolyticus (Sigma-Aldrich, Saint Louis, MO, USA, H1136-1AMP) in serum free-medium at a final concentration of 1 U/mL. After a 1 h incubation at 37 °C, 5% CO_2_, the assays were conducted with the addition of the cytostatic cytarabine (wound healing assay) or the seeding of the cells on collagen type I substrate (adhesion assay), as described above.

### 2.8. RNA Isolation, cDNA Synthesis and Real-Time PCR

MCF-7 and MDA-MB-231 cells were cultured in petri dishes at a density of 60 × 10^4^ cells for 24 h and were serum starved overnight. In order to evaluate the effects hyaluronidase in the expression of MT1-MMP, cells were first pre-treated with hyaluronidase for a 1 h incubation at 37 °C, 5% CO_2_. Then, the HA and sHA fractions were added to the cultures at a final concentration of 200 μg/mL. Following a 24 h incubation, the cells were collected, and RNA isolation was carried out using the NucleoSpin^®^ RNA II Kit (Macherey-Nagel, Allentown, PA, USA). To quantify the isolated RNA, the absorbance of each sample was measured at 260 nm and RNA purity was determined by evaluating the 260/280 nm and 260/230 nm ratios. For the cDNA synthesis, the PrimeScript™ 1st strand cDNA synthesis kit perfect real time (Takara Bio Inc., Kusatsu, Japan) was used. Real-time PCR was conducted using KAPA Taq ReadyMix DNA Polymerase (KAPA BIOSYSTEMS, Wilmington, MA, USA) according to the manufacturer’s instructions in 20 μL reaction mixture. The amplification was performed utilizing Rotor Gene Q (Qiagen, Hilden, Germany). All the reactions were performed in triplicate and a standard curve was included for each pair of primers for assay validation. In addition, a melting curve analysis was performed for detecting the SYBR Green-based objective amplicon. To provide quantification, the point of product accumulation in the early logarithmic phase of the amplification plot was defined by assigning a fluorescence threshold above the background, defined as the threshold cycle (Ct) number. Relative expression of different gene transcripts was calculated using the ΔΔCt method. The Ct of any gene of interest was normalized to the Ct of the normalizer (GAPDH). Fold changes (arbitrary units) were determined as 2^−ΔΔCt^. The genes of interest and the primers used are presented in Table 1.

### 2.9. Immunofluorescence

MCF-7 and MDA-MB-231 cells were seeded on glass coverslips in 24-well plates at a density of 6 × 10^4^ and 5 × 10^4^ cells per well respectively. The cells were cultured in complete cell culture medium for 24 h and then serum starved overnight. The next day, the HA and sHA fragments were added at a final concentration of 200 μg/mL and the cells were incubated for 24 h. Following a PBS wash, the cells were fixed in cold methanol and acetone for 5 min each. Afterwards, the cells were washed three times with PBS-Tween 0.01% and were permeabilized with 0.05% Triton X-100/PBS-Tween 0.01%. The coverslips were blocked with 5% BSA in PBS-Tween 0.01% for 1 h, and were subsequently stained with the primary antibody against e-cadherin (Takara, Kusatsu, Japan, ECCD-2, 1:200) and CD44 (Hermes-3) (Abcam, Cambridge, UK, 1:1000) in 1% BSA/PBS, overnight at 4 °C. The secondary antibody (anti-mouse Alexa Fluor-594, 1:1000) (Biotium, Fremont, CA, USA) was used in 1% BSA/PBS-Tween 0.01% for a 1-h incubation in the dark, and the coverslips were mounted with DAPI on microscope slides. The stained slides were observed through a 60× objective using a fluorescence microscope (OLYMPUS CKX41, Waltham, MA, USA) and images were captured using the QImaging MicroPublisher 3.3RTV digital camera (Adept Turnkey, Perth, Australia). Additionally, the intensity of the immunofluorescence was calculated and quantified as corrected total cell fluorescence (CTCF) with the formula: CTCF = Integrated Density— (Area of selected cell × Mean fluorescence of background readings) using image analysis software (Fiji v1.52p).

### 2.10. Western Blot Analysis

MCF-7 and MDA-MB-231 cells were seeded in petri dishes at a confluency of 90%. The cells were cultured in complete cell culture medium for 24 h and then serum starved overnight. The next day, the HA and sHA fragments were added at a final concentration of 200 μg/mL and the cells were incubated for 24 h. Following a triple wash with cold PBS, the cells were lysed using lysis buffer (25 mM HEPES, 150 mM NaCl, 5 mM EDTA, 10% glycerol, 1% Triton X-100, 0.5 mM sodium orthovanadate (Sigma-Aldrich) and protease inhibitor cocktail 1× (Chemicon, Millipore, Burlington, MA, USA) at 4 °C for 30 min. Total protein quantity was calculated using Bradford assay (Thermo Scientific, Waltham, MO, USA) and equal protein samples were reduced with β-mercaptoethanol and boiled at 100 °C for 3 min. The samples were then separated by 10% SDS-PAGE and transferred to PVDF membranes (Macherey-Nagel). The membranes were blocked with 5% BSA in TBS 0.1% Tween for 2 h at room temperature and incubated overnight with the Hermes-3 antibody (Abcam, 1:1000). The following day the membranes were incubated with HRP-conjugated secondary goat anti-mouse IgG (Sigma-Aldrich) for 2 h at room temperature and the subsequent protein detection was performed by Pierce ECL Western Blotting Substrate (Thermo Scientific), according to the manufacturer’s instructions.

### 2.11. Statistical Analysis

Reported values are expressed as mean ± standard deviation (SD) of experiments in triplicate. Statistically significant differences were evaluated using the analysis of variance (ANOVA) test followed by Tukey’s test to determine statistical differences between each data set of the three groups (control, 50 kDa HA, and sHA treatments). Differences were considered statistically significant at the level of *p* ≤ 0.05, indicated by an asterisk (*) for the treatment and control group comparison and by a hash sign (#) for the comparison between the two treatments. Statistical analysis and graphs were made using GraphPad Prism 8 (GraphPad Software, San Diego, CA, USA).

## 3. Results

### 3.1. Sulfated HA Affects Breast Cancer Cells’ Functional Properties

**Figure 1:**
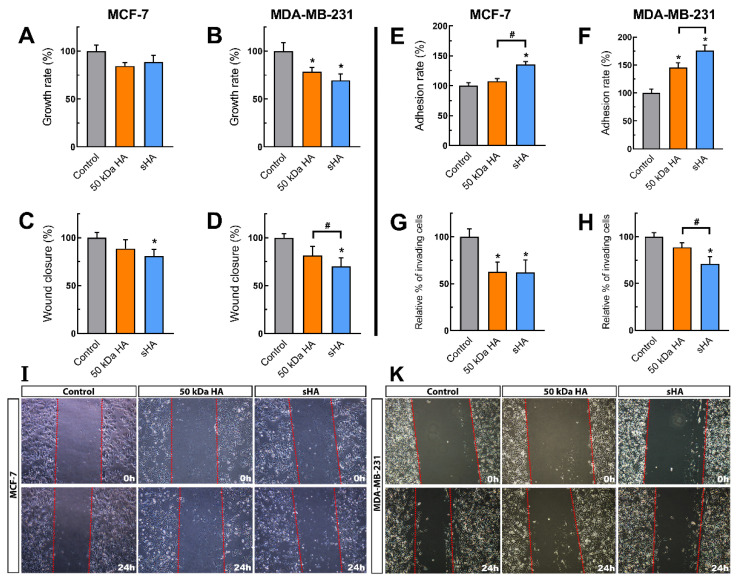
The effects of HA and sHA 50 kDa fragments on the functional properties of MCF-7 and MDA-MB-231 breast cancer cells. (**A**,**B**) Cell proliferation after 24 h of treatment at a concentration of 200 μg/mL. (**C**,**D**) Cell migration after 24 h of treatment at a concentration of 200 μg/mL. (**E**,**F**) Cell adhesion on collagen type I after 24 h of treatment at a concentration of 200 μg/mL. (**G**,**H**) Cell invasion after 24 h of treatment at a concentration of 200 μg/mL. Each bar represents mean ± SD values from triplicate samples. (**I**,**K**) Photos of the wound healing assays at time point 0 and 24 h. An asterisk (*) indicates statistically significant differences (*p* < 0.05) compared to the control groups and (#) indicates statistically significant differences (*p* < 0.05) between the treatments.

In order to evaluate the effects of sHA on the ERα-positive MCF-7 and the ERα-negative/ERβ-positive MDA-MB-231 cell lines, the cells were treated with non-sulfated HA and sHA fragments of 50 kDa at a final concentration of 200 μg/mL, based on a previous study of our research group [25]. The first step was to determine whether sHA affects cell proliferation. The obtained results indicate a slight decrease in the proliferation capability of the MCF-7 cells for both HA fragments tested (Figure 1A). On the other hand, the MDA-MB-231 cell proliferation exhibits a significant decrease, both following treatment with the sHA fragments (ca 31%) and the non-sulfated 50 kDa HA (ca 22%) (Figure 1B). 

A hallmark of cancer progression is the ability of cancer cells to migrate and metastasize [35]. For this reason, the migration, adhesion, and invasive properties of the breast cancer cells were further investigated. Notably, treatment with sHA results in lower migratory capacity for both MCF-7 (ca 20%) and MDA-MB-231 cells (ca 30%), while non-sulfated HA of the same size do not exhibit a similar effect (Figure 1C,D,I,K). Next, the adhesiveness of the cells on collagen type I was determined. According to the obtained data, treatment with sHA leads to an increase of ca 35% for the MCF-7 (Figure 1E) and ca 76% for the MDA-MB-231 cells (Figure 1F). Lastly, the effects of cellular invasion on collagen type I were investigated. Both the non-sulfated and sHA fragments show a decreased the invasiveness (ca 38%) of the MCF-7 cells (Figure 1G), whereas in the MDA-MB-231 cells, the invasiveness is affected significantly only after the sHA treatment, with a decrease of ca 30% (Figure 1H), indicating that the various effects of sHA depend on the breast cancer cell types bearing different ERs among other biomolecules.

### 3.2. Sulfated HA Competition with Endogenous HA Affects Cell Migration and Adhesion

**Figure 2:**
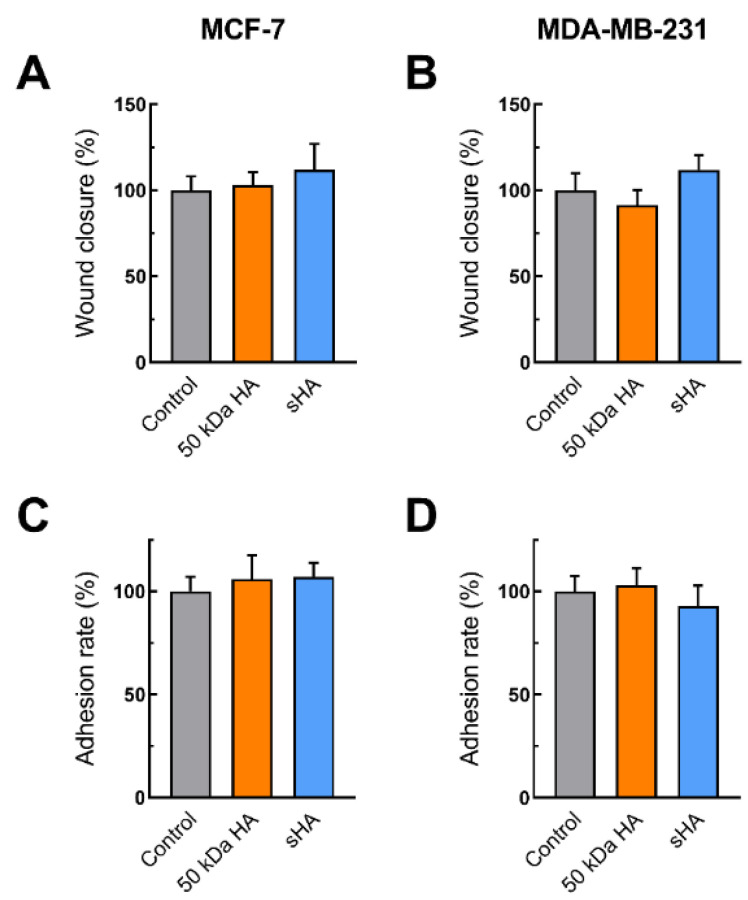
The effects of HA and sHA 50 kDa fragments on the migratory and adhesive potential of MCF-7 [(**A**,**C**), respectively] and MDA-MB-231 [(**B**,**D**), respectively] following treatment with hyaluronidase at a final concentration of 1 U/mL for 1 h. Each bar represents mean ± SD values from triplicate samples.

As shown above, the sHA fragments significantly attenuate the migratory potential of MDA-MB-231 cells and slightly the MCF-7 ones. To investigate whether this effect could be attributed to competition between the exogenously added sHA fragments and the endogenous HA, the breast cancer cells were pre-treated with hyaluronidase as to remove the existing pericellular HA coating, and a wound healing assay following treatments with HA and sHA treatments was subsequently performed. Notably, the obtained data show an alteration of the previously observed effects, with no significant changes appearing in the migratory capabilities in both cell lines following hyaluronidase treatment (Figure 2A,B). To further evaluate whether hyaluronidase could affect adhesion, another major functional property, the same setup with hyaluronidase was applied. It is noted that no significant differences were observed upon such a treatment (Figure 2C,D). It is therefore plausible to suggest that the effects of sHA fragments on the migratory as well as the adhesive potential of both breast cancer cell lines could at least in part be attributed to competition with endogenous HA.

### 3.3. Sulfated HA Modulates the Expression of EMT Markers

**Figure 3:**
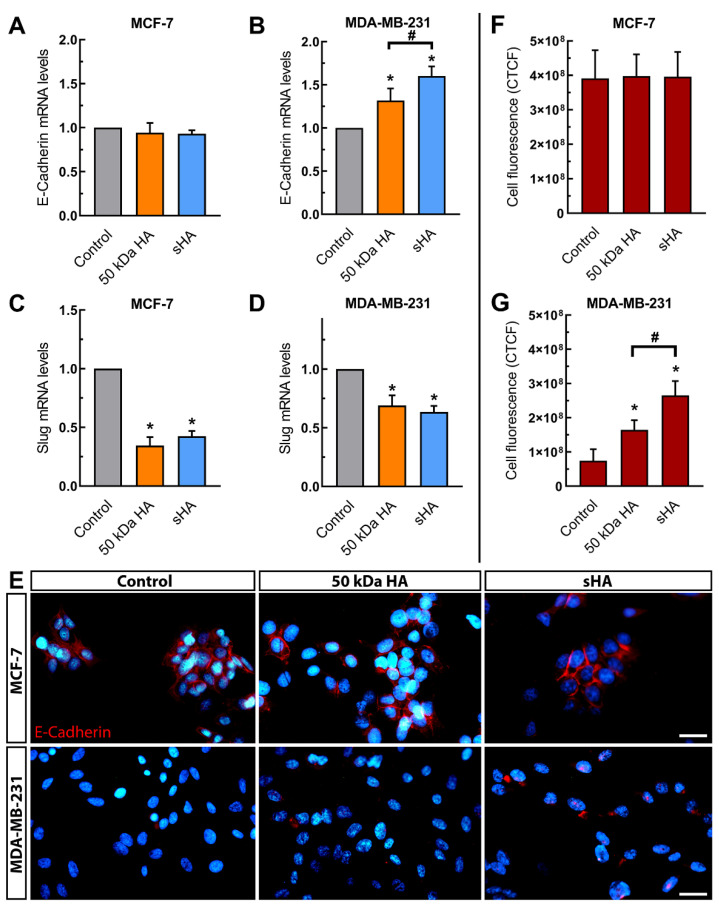
The effects of HA and sHA 50 kDa fragments on the expression of EMT markers. (**A**,**B**) Quantitative RT-PCR analysis of e-cadherin mRNA levels in MCF-7 (**A**) and MDA-MB-231 cells (**B**) following a 24 h treatment at a final concentration of 200 μg/mL. (**C**,**D**) Quantitative RT-PCR analysis of snail2/slug mRNA levels following a 24 h treatment at a final concentration of 200 μg/mL in MCF-7 (**C**) and MDA-MB-231 cells (**D**). (**E**) Immunofluorescence imaging for e-cadherin (red) after treatment with HA and sHA fragments for 24 h at a final concentration of 200 μg/mL. Nuclei are shown in blue (DAPI). Scale bars: 20 μm. (**F**,**G**) Quantification of e-cadherin fluorescence, calculated as corrected total cell fluorescence (CTCF) in both breast cancer cell lines following treatment. Each bar represents mean ± SD values from triplicate samples. An asterisk (*) indicates statistically significant differences (*p* < 0.05) compared to the control groups and (#) indicates statistically significant differences (*p* < 0.05) between the treatments.

A vital process in cancer progression is the EMT, during which epithelial markers such as e-cadherin are suppressed, while mesenchymal markers such as snail2/slug are elevated [8]. For this reason, the potential implication of sHA in the expression of EMT markers was investigated. Treatment with either HA or sHA fragments resulted in upregulation of e-cadherin of the mesenchymal-like MDA-MB-231 cells, with non-sulfated HA yielding a 1.3-fold change in the mRNA levels of the marker and sHA showing a 1.6-fold change (Figure 3B). On the other hand, no differences are observed in the mRNA levels of e-cadherin for the epithelial-like MCF-7 cells following the same treatment (Figure 3A). The above results are further corroborated by immunofluorescence for the e-cadherin protein (Figure 3E) and its subsequent intensity quantification analysis, calculated as corrected total cell fluorescence (CTCF) (Figure 3G,F). Additionally, the expression levels of the mesenchymal marker snail2/slug were studied. Following treatment with non-sulfated and sHA a significant downregulation of snail2/slug in the MCF-7 cells was observed, both by non-sulfated HA (ca 66%) and sHA (ca 60%) (Figure 3C). Similarly, the mRNA levels of snail2/slug are also diminished after treatments, with non-sulfated HA and sHA causing a significant downregulation (ca 31% and 37%, respectively) (Figure 3D).

### 3.4. Sulfated HA Downregulates the Expression of Matrix Metalloproteinases

**Figure 4:**
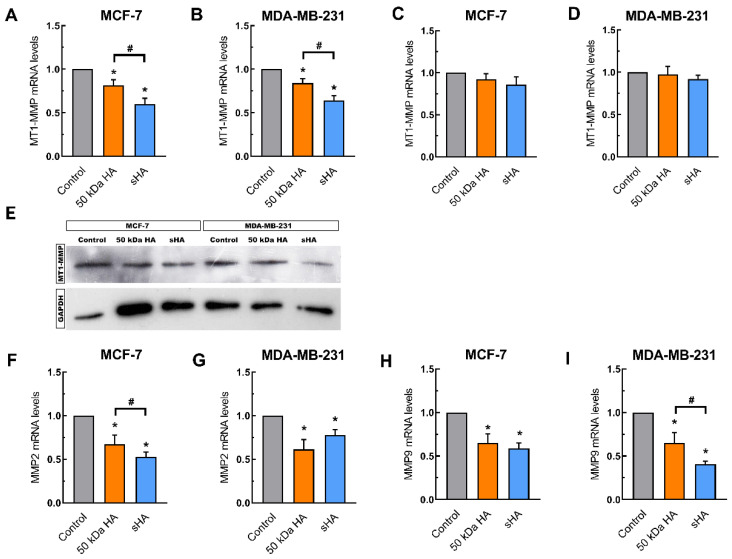
The effects of HA and sHA 50 kDa fragments on the expression of matrix remodeling enzymes. (**A**,**B**) Quantitative RT-PCR analysis of MT1-MMP mRNA levels in MCF-7 (**A**) and MDA-MB-231 cells (**B**) following a 24 h treatment at a final concentration of 200 μg/mL. (**C**,**D**) Quantitative RT-PCR analysis of MT1-MMP mRNA levels following hyaluronidase pre-treatment in MCF-7 (**C**) and MDA-MB-231 cells (**D**). (**E**) Western blot analysis of MT1-MMP protein levels in MCF-7 and MDA-MB-231 cells. (**F**,**G**) Quantitative RT-PCR analysis of MMP2 mRNA levels following a 24 h treatment at a final concentration of 200 μg/mL in MCF-7 (**F**) and MDA-MB-231 cells (**G**). (**H**,**I**) Quantitative RT-PCR analysis of MMP9 mRNA levels following a 24 h treatment at a final concentration of 200 μg/mL in MCF-7 (**H**) and MDA-MB-231 cells (**I**). Each bar represents mean ± SD values from triplicate samples. An asterisk (*) indicates statistically significant differences (*p* < 0.05) compared to the control groups and (#) indicates statistically significant differences (*p* < 0.05) between the treatments.

To further evaluate the changes caused by sHA in the functional properties of the breast cancer cells, the expression of matrix metalloproteinases was studied. MMPs are enzymes involved in ECM remodeling and are found significantly modulated in breast cancer. Firstly, the expression of MT1-MMP, which has been linked with migration and cell invasion in breast cancer cells, was investigated [23]. Treatment with non-sulfated HA and sHA significantly decreased the mRNA levels of MT1-MMP in both breast cancer cell lines. For the MCF-7 cells a decrease of ca 20% and ca 40% is caused by the non-sulfated HA and sHA fragments, respectively (Figure 4A). In MDA-MB-231 cells these suppressive effects were more profound (31 and 47%, respectively) (Figure 4B). These data were further confirmed at the protein level using Western blotting (Figure 4E). It is worth noting that the observed effects were abolished using pre-treatment with hyaluronidase, demonstrating the importance of competition between the added HA and sHA fragments with the endogenous HA (Figure 4C,D), even in the more profound inhibitory effect exerted by sHA.

Moreover, the expression of MMP2 and MMP9, two key enzymes in ECM degradation, was also investigated [11]. The mRNA levels of MMP2 are significantly altered upon treatment with fragments, with non-sulfated HA causing downregulation in both MCF-7 (ca 33%) and MDA-MB-231 cells (ca 40%). sHA decreased the levels of MMP2 in the respective cell lines ca 48% and ca 23% (Figure 4F,G). Following a similar pattern, the expression levels of MMP9 were also downregulated after treatment, with non-sulfated HA (ca 35% decrease in both MCF-7 and MDA-MB-231 cells), while the sHA fragments caused a more profound decrease (ca 42% and ca 60% in MCF-7 and MDA-MB-231, respectively) (Figure 4H,I).

### 3.5. Sulfated HA Modulates the Expression of HAS but Not CD44

**Figure 5:**
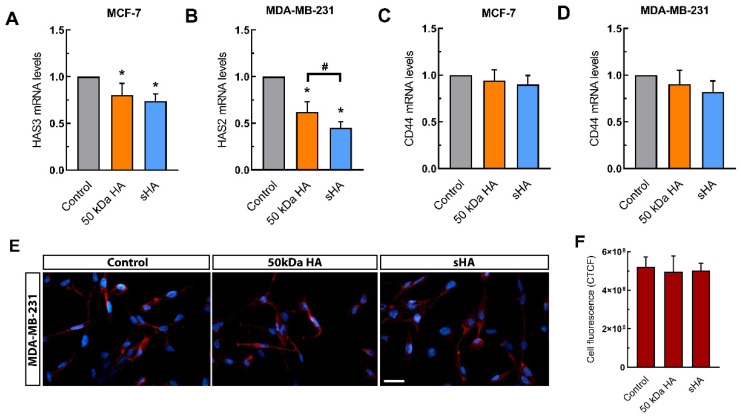
The effects of HA and sHA 50 kDa fragments on the expression of HA partners. (**A**,**B**) Quantitative RT-PCR analysis of HAS3 mRNA levels in MCF-7 (**A**) and HAS2 mRNA MDA-MB-231 cells (**B**) following a 24 h treatment at a final concentration of 200 μg/mL. (**C**,**D**) Quantitative RT–PCR analysis of mRNA levels of CD44 mRNA levels following a 24 h treatment at a final concentration of 200 μg/mL in MCF-7 (**C**) and MDA-MB-231 cells (**D**). (**E**) Immunofluorescence imaging for CD44 (red) after treatment with HA and sHA fragments for 24 h at a final concentration of 200 μg/mL. Nuclei are shown in blue (DAPI). Scale bars: 20 μm. (**F**) Quantification of CD44 fluorescence, calculated as corrected total cell fluorescence (CTCF) in MDA-MB-231 cells. Each bar represents mean ± SD values from triplicate samples. An asterisk (*) indicates statistically significant differences (*p* < 0.05) compared to the control groups and (#) indicates statistically significant differences (*p* < 0.05) between the treatments.

Considering the effects sHA of on the breast cancer cells’ functional properties, the correlation between these changes and modulations to the expression of HA partners, such as HA synthases (HAS2 and -3) and CD44, was investigated. HAS2 is the main enzyme responsible for HA synthesis in MDA-MB-231 cells and its elevated levels have been linked to EMT, invasive potential and metastasis, whereas HAS3 is the main HA active synthase in MCF-7 cells [36,37,38,39]. Our data indicate that treatments with non-sulfated or sHA fragments lead to significant downregulation of HAS3 and HAS2 in MCF-7 and MDA-MB-231 cells, respectively. Specifically, non-sulfated HA causes a decrease in HAS3 mRNA levels of ca 20%, and sHA decreases HAS-3 expression by ca 30% (Figure 5A). HAS2 mRNA levels in MDA-MB-231 cells were significantly downregulated by ca 38% upon treatment with non-sulfated HA and ca 65% upon treatment with sHA fragments (Figure 5B).

CD44 is one of the main HA receptors, and its overexpression correlates with cell migration, EMT and metastasis in breast cancer [18,40]. Therefore, we evaluated whether sHA plays a role in altering the expression of CD44. Following a 24 h treatment with 200 μg/mL HA and sHA in MCF-7 and MDA-MB-231 cells, no significant differences were observed in the expression (mRNA and protein) levels of CD44, except a slight decrease seen upon treatments with sHA, in both breast cancer cell line tested (Figure 5C–F). These data indicate that CD44 is sufficiently available for the extra signaling or other receptors may be implicated.

## 4. Discussion

HA is a major macromolecule of the three-dimensional ECM network, playing a crucial role in a variety of biological processes, including cancer development and progression. Through its binding to the receptor CD44, HA regulates intracellular signaling pathways and cell functional properties in a size-dependent manner [26]. Recent studies have shown the role of HA fragments of different molecular size in modulating breast cancer cells proliferation, migration, and invasion [24,25]. Furthermore, treatment with such HA fragments results in significant alterations in the morphology and aggressive phenotype of breast cancer cells. HA is the only GAG not physically modified by the addition of sulfate groups [13]. Research studies, however, have previously demonstrated the potential of sHA fragments to exhibit antitumor effects on prostate and bladder cancer cells [30,31]. In the present study, we investigated the effects of sHA on breast cancer cells with different ER status; the low metastatic ERα-positive MCF-7 and the more aggressive ERα-negative/ERβ-positive MDA-MB-231 cells. To this end, non-sulfated HA and the sHA fragment of the same molecular weight (50 kDa) were evaluated regarding their effects on the breast cancer cell functional properties. Moreover, the expression of key ECM effectors was also studied.

Our data demonstrate that treatment with sHA leads to an attenuation of breast cancer cell proliferation, migration, and invasion, while also increasing cell adhesion on collagen type I. Notably, sHA exhibited a more profound effect compared to the non-sulfated HA counterpart of the same molecular size, thus suggesting an anticancer regulatory capability for sHA. Additionally, differences in the effects of sHA between the two breast cancer cell lines suggest the dependency of its action on the type of cells, among other parameters, the ER status is considered a key difference. These observations are further supported by modulations to the expression of matrix metalloproteinases, which are typically overexpressed during breast cancer progression. Treatment with sHA, however, lead to the downregulation of MT1-MMP, known to be involved in breast cancer cell migration and invasion, as well as the downregulation of MMP2 and MMP9, two MMPs with pivotal role in ECM degradation [23]. Additionally, sHA caused a significant downregulation in the expression of HA synthases, which have been linked to breast cancer invasion and metastasis [36,37,38,39].

In the study by Li et al. [41], the authors demonstrated that the CD44-mediated TGF-β1 and EGF signaling, and the co-localization of CD44/EGFR influenced the activation of EGF signaling induced by TGF-β1 in lung and breast cancer cells. The effects were abolished following inhibition of HAS2 by 4-MU. The 50 kDa sHA effects, as clearly demonstrated here, may well be at least in part attributed to the competition with the endogenous HA.

An important process aiding in breast cancer metastasis is the epithelial-to-mesenchymal transition [7]. Our findings show that sHA alters the expression of EMT markers, causing an upregulation of the epithelial e-cadherin for the mesenchymal-like MDA-MB-231 cells and downregulating the mesenchymal marker snail2/slug in both breast cancer cell lines. Taking these changes in EMT markers expression into account, it would be of high interest to examine whether sHA also affects the cell morphology, using scanning electron microscopy. Based on the results obtained for the functional properties (migration and adhesion) as well as in the expression of MT1-MMP using hyaluronidase, it is plausible to suggest that the effects of sHA fragment could at least in part be attributed to competition with endogenous HA. Adding to this, no significant changes in the expression of CD44 was found following sHA treatment, possibly since the CD44 is sufficiently available and/or other changes in intracellular signaling pathways play a role. This, however, requires further elucidation.

In conclusion, our findings demonstrate that sHA regulates breast cancer cells’ functional properties and the gene expression profile of ECM effectors. The exact mechanism by which the sHA fragments drive these actions has to be further elucidated in the future. Consequently, our novel data open new avenues for further research in respect to breast cancer targeting and designate sHA as a useful molecule in the development of therapeutic strategies.

## Figures and Tables

**Table 1:** Primer sequences used for the genes of interest in real-time PCR.

Gene		Primer Sequence (5′–3′)	Annealing T (°C)
HAS2	F	TCGCAACACGTAACGCAAT	60 °C
	R	ACTTCTCTTTTTCCACCCCATTT	
HAS3	F	AACAAGTACGACTCATGGATTTCCT	60 °C
	R	GCCCGCTCCACGTTGA	
e-cadherin	F	TACGCCTGGGACTCCACCTA	60 °C
	R	CCAGAAACGGAGGCCTGAT	
snail2/slug	F	AGACCCTGGTTGCTTCAAGGA	60 °C
	R	CTCAGATTTGACCTGTCTGCAAA	
MT1-MMP	F	CATGGGCAGCGATGAAGTCT	60 °C
	R	CCAGTATTTGTTCCCCTTGTAGAAGTA	
MMP2	F	CGTCTGTCCCAGGATGACATC	62 °C
	R	ATGTCAGGAGAGGCCCCATA	
GAPDH	F	AGGCTGTTGTCATACTTCTCAT	60 °C
	R	GGAGTCCACTGGCGTCTT	
MMP9	F	TTCCAGTACCGAGAGAAAGCCTAT	60 °C
	R	GGTCACGTAGCCCACTTGGT	
CD44	F	ATAATAAAGGAGCAGCACTTCAGGA	60 °C
	R	ATAATTTGTGTCTTGGTCTCTGGTAGC	

## Data Availability

The data presented in this study are available on request.
